# Biomarkers of Hypochromia: The Contemporary Assessment of Iron Status and Erythropoiesis

**DOI:** 10.1155/2013/603786

**Published:** 2013-02-28

**Authors:** Eloísa Urrechaga, Luís Borque, Jesús F. Escanero

**Affiliations:** ^1^Laboratory Hospital Galdakao-Usansolo, 48960 Galdakao, Biscay, Spain; ^2^Department of Pharmacology and Physiology, Faculty of Medicine, University of Zaragoza, 50009 Zaragoza, Spain

## Abstract

Iron status is the result of the balance between the rate of erythropoiesis and the amount of the iron stores. Direct consequence of an imbalance between the erythroid marrow iron requirements and the actual supply is a reduction of red cell hemoglobin content, which causes hypochromic mature red cells and reticulocytes. The diagnosis of iron deficiency is particularly challenging in patients with acute or chronic inflammatory conditions because most of the biochemical markers for iron metabolism (serum ferritin and transferrin ) are affected by acute phase reaction. For these reasons, interest has been generated in the use of erythrocyte and reticulocyte parameters, available on the modern hematology analyzers. Reported during blood analysis routinely performed on the instrument, these parameters can assist in early detection of clinical conditions (iron deficiency, absolute, or functional; ineffective erythropoiesis, including iron restricted or thalassemia), without additional cost. Technological progress has meant that in recent years modern analyzers report new parameters that provide further information from the traditional count. Nevertheless these new parameters are exclusive of each manufacturer, and they are patented. This is an update of these new laboratory test biomarkers of hypochromia reported by different manufactures, their meaning, and clinical utility on daily practice.

## 1. Advances in Basic Science

The control of iron homeostasis acts at both the cellular and the systemic level and involves a complex system of different cell types, transporters, and signals. To maintain systemic iron homeostasis, communication between cells that absorb iron from the diet (duodenal enterocytes), consume iron (mainly erythroid precursors), and store iron (hepatocytes and tissue macrophages) must be tightly regulated [1].

In the last 10 years, understanding of the regulation of iron homeostasis has changed substantially. A small peptide hormone, hepcidin, emerged as the central regulator of iron absorption, plasma iron levels, and iron distribution. The hormone controls the iron homeostasis and acts by inhibiting iron flows into plasma from macrophages involved in recycling of senescent erythrocytes, duodenal enterocytes engaged in the absorption of dietary iron, and hepatocytes that store iron [2].

The same factors that influence dietary iron absorption, that is, iron stores, erythropoietic activity, hemoglobin, oxygen content, and inflammation, also regulate the expression of hepcidin by hepatocytes.

Hepcidin reduces the quantity of circulating iron by limiting the egress of the metal from both intestinal and macrophage cells and is the key for the regulation of systemic iron homeostasis [3].

## 2. Iron Status

Absolute iron deficiency is defined as a decreased total iron body content. Iron deficiency anemia (IDA) occurs when iron deficiency is sufficiently severe to diminish erythropoiesis and cause the development of anemia.

Distinction between IDA and other entities in the differential diagnosis of anemia, especially anemia that accompanies infection, inflammation, and cancer, commonly termed anemia of chronic disease (ACD), to distinguish ACD from the combined state IDA/ACD is a daily challenge for clinicians and laboratory professionals [4].

It has long been known that inflammation can mimic some aspects of iron deficiency by impairing the utilization of existing iron stores for red cell production and inducing an iron-sequestration syndrome and low serum iron. The molecular mechanisms that underlie the redistribution of iron during inflammation center on the cytokine-stimulated overproduction of hepcidin.

Functional iron deficiency describes a state where the total iron content of the body is normal or even elevated, but the iron is “locked away” and unavailable for the production of red blood cells. Iron becomes limiting for erythropoiesis, but generally the resulting anemia is not severe [2].

The laboratory diagnosis of absolute iron deficiency has been based on low serum iron, low percent transferrin saturation (TSAT), and low ferritin [5]. The limitations of using transferrin saturation reflect those of serum iron, that is, wide diurnal variation and low specificity. It is also reduced in inflammatory disease [6, 7].

On the other hand, as ferritin is an acute phase reactant, its serum levels may be elevated in the presence of chronic inflammation, infection, malignancy, and liver disease, making ferritin somewhat less than an ideal test for determining iron deficiency [8].

Serum transferrin receptor (sTfR) is not affected by inflammation [9] which would make sTfR a more reliable test than serum ferritin when inflammation is present. sTfR and the derived sTfR/log ferritin (ferritin index) are reliable markers of iron deficiency in mixed situations [10].

A particular case of ACD is represented by anemia of chronic kidney disease (CKD). Anemia is one of the most characteristic manifestations of CKD. The most well-known cause is inadequate production of erythropoietin, but there are also other causes leading to impaired erythropoiesis (reduced proliferative activity of erythroid precursors in bone marrow, reduced survival of red cells, and decreased iron availability), contributing to anemia [11, 12].

## 3. The Clinician's Need for Reliable Laboratory Tests

Recombinant human erythropoietin (rHuEpo) for the treatment of CKD and patients with anemia related to cancer has been available since 1989. However, rHuEpo therapy results in functional iron deficiency due to insufficient iron stores for the accelerated erythropoiesis [13].

Iron deficiency is the main cause of suboptimal response to erythropoietin in dialysis patients. Maintenance iron supplementation is required to successfully treat anemia. Long-term orally administered iron therapy is limited by noncompliance, gastrointestinal side effects, insufficient absorption, and drug interaction; intravenous iron compounds are used to treat dialysis patients who become iron deficient [14].

Monitoring erythropoietin treated patients' iron status is important to detect iron deficiency and avoid the adverse effects of iron medication [15].

The most important question regarding anemia therapy in these patients is which are the best parameters to assess the iron available for erythropoiesis, and the need for predictors and indicators of effectiveness has not abated [16, 17]. 

Interest has been generated in the use of erythrocyte and reticulocyte new indices available on the modern analyzers based on flow cytometry technology. 

In 2004, European Best Practice Guidelines suggested an hemoglobin (Hb) target of 110 g/L [18]; the assessment of anemia in CKD patients should include the laboratory measurement of the following parameters:

- Hb concentration, to assess the degree of anemia;
- red blood cell indices (mean cell volume MCV, mean cell hemoglobin MCH), to assess the type of anemia;
- absolute reticulocyte count, to assess erythropoietic activity;
- serum ferritin concentration, to assess iron stores;
- functional iron available for erythropoiesis:
  - percentage of hypochromic red cells (Siemens);
  - transferrin saturation;
  - reticulocyte hemoglobin content (Siemens);
- plasma C-reactive protein, to assess inflammation.

Two parameters are exclusive of one-manufacturer's counters.

Direct consequence of an imbalance between the erythroid marrow iron requirements and the actual supply is a reduction of red cell hemoglobin content, which causes hypochromic mature red cells and reticulocytes. The modern hematological parameters contribute to the advanced study of the anemia and depend on the technology employed; the debate about other parameters with the same clinical meaning and potential utility as reticulocyte hemoglobin content and percentage of hypochromic red cells reported by Siemens analyzers is open.

## 4. The Advance of Technology 

The hemogram is one of the more required tests by the clinicians, the analysis nowadays is totally automated, and the correct interpretation of the results requires to reunite the knowledge about the characteristics of the equipment and the clinical meaning of the results. The suppliers contribute innovations, providing new parameters that can help the clinicians to make a diagnosis in a fast, cheap, and useful manner [19].

The professionals of the clinical laboratory must obtain the maximum yield of the new technologies obtaining as much information as possible. 

Automated blood cell counters have changed substantially during the last 20 years. Technological progress has meant that in recent years modern analyzers, fully automated, have been available. These analyzers report new parameters that provide further information from the traditional count; this information must be evaluated to prove the potential clinical utility in different clinical situations.

Each company applies the technology in a different way in the analyzers, and for this reason these new parameters are exclusive of each manufacturer, and they are patented.  Table 1 summarizes the RBC extended parameters now reported by the different analyzers.

Flow cytometry provides information about individual cell characteristics. This is in contrast to previous measurements of MCV, MCH, and MCHC which only calculate mean values for the total red cell population. 

Modern counters can provide information about the reticulocyte counts but also about the characteristics of these cells (size or Hb content), related to the quality of the erythropoiesis, giving information of the current erythropoietic activity of the bone marrow. 

Reticulocytes are immature red blood cells with a life span of only 1 to 2 days. When these are first released from the bone marrow, measurement of their hemoglobin content or volume can reflect the amount of iron immediately available for erythropoiesis. 

Red blood cells (RBCs) are continuously produced in the bone marrow; when a state of iron deficiency proceeds and the iron stores progressively decrease, mean cell volume (MCV), mean cell hemoglobin (MCH), and red blood cell count (RBC) tend to decline. In iron deficient erythropoiesis, synthesis of Hb molecules is severely impaired leading to the production of erythrocytes with low Hb concentration (hypochromic cells). Because of their long-life span of approximately 3 months, several cohorts of normochromic and increasingly hypochromic red cells coexist in the peripheral blood leading to anisocytosis; red cell distribution width (RDW) reflects the variation of size of the red cells.

Anisocytosis and anisochromia are related concepts but not identical; RBCs undergo a rapid reduction in volume and hemoglobin in the few days after release from the bone marrow; in healthy individuals (and patients with mild disease) the characteristics of the population can be stable. But in clinical setting such as IDA the production of reticulocytes appears to be counterbalanced by delayed clearance of old cells, and the natural evolution of the red cells is altered [20]. 

MCV is the mean of the volumes of all erythrocytes; RDW refers to the variety of volumes present in the red cell population, so the contribution of marginal-sized subsets to the calculated mean value can be assessed.

This is not the case for Hb content. MCH is calculated from red blood cell count and Hb and represents the average; the percentage of RBC subsets can give complementary information of the contribution of cell with extreme values of Hb (hypochromic and hyperchromic cells) to the mean values, reflecting the fluctuations of iron availability to the erythron in the previous weeks.

The assessment of the erythrocyte subsets is an added information can be useful, as it reflects the situation of the whole of RBC.

## 5. Siemens (Siemens Healthcare Diagnostics, Deerfield, IL, USA)

Applying flow cytometry technology, the detection of two light-scatter signals allows the independent measurements of cell-by-cell characteristics of volume and hemoglobin concentration; on the reticulocyte channel of ADVIA analyzers immature red cells are stained based on their RNA content using the stain Oxazine 750.

The hypochromic red cells (referred to as %Hypo) and CHr (reticulocyte hemoglobin content) are reported by the Siemens ADVIA 120 hematology analyzer (Siemens Medical Solutions Diagnostics, Tarrytown, NY, USA). Reticulocyte Hb content (CHr) and the percentage of hypochromic red blood cells (%Hypo) reflect iron availability and are reliable markers of functional iron deficiency [21, 22].

The measurement of CHr is a direct assessment of the incorporation of iron into Hb and thus an estimate of the recent functional availability of iron to the erythron; due to the life span of the reticulocytes CHr is a sensitive indicator of iron deficient erythropoiesis, cutoff 28-29 pg [23, 24].

rHuEpo is effective in stimulating production of red blood cells, but without an adequate iron supply to bind to heme, and the red blood cells will be hypochromic, low in Hb concentration. Thus, in states of iron deficiency, a significant percentage of red blood cells leaving the bone marrow will have a low Hb concentration. By measuring the percentage of RBCs with Hb concentration <280 g/L, iron deficiency can be detected. Hypochromic red cells percentages have been correlated with iron deficiency. %Hypo is reported by Siemens Advia 120 hematology analyzer based on the optical cell-by-cell hemoglobin measurement. 

The measurement of %Hypo (defined as the percentage of red blood cells with Hb concentration less than 280 g/L) is a sensitive method for quantifying the hemoglobinization of mature red cells. Because of the long circulating life span of mature erythrocytes %Hypo values are related to iron status in the last 2-3 months and have been recognised as an indicator of iron deficiency [25–27]. %Hypo < 5% is considered normal. Two different criteria, more specifically, %Hypo > 5% and >10% have been used. %Hypo > 10%, has been more commonly used for defining absolute iron deficiency and functional iron deficiency [18].

CHr and %Hypo have been used as a diagnostic tool, together with biochemical markers, to distinguish IDA from ACD, and are incorporated to the guidelines for the monitoring of recombinant human erythropoietin rHuEpo therapy [18, 28–31]. These red blood cell and reticulocyte indices have also been recognized as reliable markers of iron deficiency on physiological conditions, where the demand for iron increases (children, women at child bearing age, pregnancy) [27, 32, 33]. 

The transition from the normal iron-replete state to the development of iron deficiency anemia is a sequential process: depletion of the storage iron compartment, followed by its exhaustion and the consequent initiation of depletion of the functional iron compartment. Normal Hb level does not exclude lack of iron storage, because individuals with normal body iron stores must lose a large amount of body iron during a long period before the Hb falls [34].

Nonanemic iron deficiency is sometimes termed “latent iron deficiency” or “depleted iron stores.” Reticulocyte-derived parameters can be useful in this context because they give information regarding the actual iron supply and the quality of the erythropoiesis within 2 days.

CHr could be a sensitive marker to detect early the negative balance between body iron content and the demand for erythropoiesis, before the mature cell indices or even Hb move below the reference intervals, improving the diagnostic algorithms [35–38]. 

The size of iron stores in blood donors can be also evaluated with confidence by means of CHr [39–41].

## 6. Abbott (Abbott Diagnostics, Santa Clara, CA, USA)

The flow-cytometric optical technology for RBC parameters measurement was first made available by the Technicon Company in their H* series of instruments, later followed by the Advia hematology analyzers (Bayer Diagnostics, presently Siemens Healthcare Diagnostics, Deerfield, IL, USA). As a consequence, many of the data reported in the literature have been generated using these analyzers. In 2010, Abbott Diagnostics introduced extended RBC parameters on the CELL-DYN Sapphire analyzer. The technology used in this instrument is multiangle laser light scattering by single cell, sphered RBC, and relies on the Mie theory, like what the Advia analyzers do. Therefore, it was anticipated that there would exist a very high level of agreement between the extended RBC parameters of both types of analyzer. Although the parameters measured are identical, the manufacturers use slightly different nomenclature (Table 1).

Mie theory describes the mathematics of light diffraction by spherical objects; in this case the red cells are transformed into isovolumetric spheres. When using monochromatic (laser) light, diffraction is only a function of the size and the refractive index of the object (related to its internal structure).

CELL-DYN Sapphire extended RBC parameters are produced collecting data of light in 3 different angles and in the reticulocyte channel of the fluorescence signal. 

Mathematical models in the software use these scatter signals for calculating, for each individual cell, estimates of cellular volume (*V*) and cellular Hb concentration (CHC); these parameters can be specifically calculated for reticulocytes, too mean cellular volume of reticulocytes (MCVr) and mean cellular Hb content of reticulocytes (MCHr).

The whole RBC population can be classify considering the Hb concentration:

 %HPO is the percentage of hypochromic RBC with CHC < 280 g/L,
 %HPR the percentage the percentage of hyperchromic RBC, CHC > 410 g/L.

It has been published the new extended RBC parameters as measured on CELL-DYN Sapphire show a high degree of correlation with those of the Advia analyzers, although the absolute values may differ [42, 43]. This renders it necessary to establish instrument-specific reference ranges and clinical decision values [44, 45].

The reference intervals for %HPO and MCHr have been established, 0–4.8% and 28.5–34.5 pg, respectively [44], and the median in patients with no anemia (2.7% for %HPO and 29.9 pg for MCHr), are already published [43].

Although the numerical values do differ from the Advia parameters because of differences in technology, their clinical utility seems to be rather comparable [43].

## 7. Sysmex (Sysmex Corporation, Kobe, Japan)

Sysmex XE analyzers (Sysmex Corporation, Kobe, Japan) employ flow cytometry technology. In the reticulocyte channel blood cells are stained by a polymethine dye, specific for RNA/DNA. A bi-dimensional distribution of forward scattered light and fluorescence is presented as a scattergram, indicating mature red cells and reticulocytes. Forward scatter correlates with erythrocyte and reticulocyte Hb content, the so-called RBC Hb equivalent (RBC He), reticulocyte Hb equivalent (Ret He).

Measurements of Ret He provide useful information in diagnosing anemia, iron-restricted erythropoiesis, and functional iron deficiency and response to iron therapy during rHuEpo therapy [46–49]; Ret He, generated by all Sysmex XE analyzers (Sysmex Corporation, Kobe, Japan), has been recognised as a direct assessment of the incorporation of iron into erythrocyte Hb and a direct estimate of the recent functional availability of iron, thus provides the same information as CHr [50–52]. Twenty-nine pg is the cutoff value that defines deficient erythropoiesis. Ret He correlates with CHr with the same clinical meaning [53–55].

The role inflammation impairing the utilization of existing iron stores has been explained previously; recent articles focus on critically ill patients and the attempts to introduce Ret He in the transfusion area improving the management of anemic patients in critical conditions [56, 57]. The iron sequestration in the macrophages could be an evolutionary mechanism of defense against determined pathogens of high virulence [1]; impairment of reticulocyte Hb content can be considered as a consequence of infection and activation of mechanisms of immunity [58].

Derived from RBC Hb equivalent (RBC He), the Sysmex XE 5000 analyzer reports the percentages of hypochromic red cells; %Hypo He indicates the percentage of hypochromic red cells with an Hb content less than 17 pg. Reference intervals are already published [59]. 

Recent studies confirm the clinical reliability of %Hypo He as marker of iron availability [60] and the assessment of functional iron deficiency in hemodialysis patients [61]; 2.7% is the cutoff value which defines iron deficiency in those patients [62].

## 8. Beckman Coulter (Beckman Coulter Inc. Miami, FL, USA)

Beckman Coulter (Beckman Coulter Inc. Miami, FL, USA) applies the Volume Conductivity Scatter technology to this field and new parameters are now available on the LH series analyzers.

Low hemoglobin density (LHD%) derives from the traditional mean cell hemoglobin concentration (MCHC), using the following mathematical sigmoid transformation:

(1) $$\begin{array}{c} \text{LHD}\%=100*\sqrt{1-\left(\frac{1}{1+e^{1.8(30-\text{ MCHC})}}\right)}. \end{array}$$

MCHC is an inclusive measure of both the availability of iron over the preceding 90–120 days and of the proper introduction of iron into intracellular Hb. In the same way LHD% is related to iron availability and the Hb content of the mature red cells.

In this equation defining LHD%, in addition to the standard sigmoid function, a square root is applied to further enhance numerical resolution in the lower end region, to improve the differentiation between the normal and the abnormal among the blood samples having relatively low values of LHD%. 

The reference range and the values of LHD% in normal population have been established, 1.0–4.0%, and the correlation with %Hypo values and its clinical usefulness in the study of iron status have been assessed [63].

LHD% is a reliable parameter for the detection of iron deficiency in patients with anemia in the presence of inflammation [64], recognizes subsets of patients, and therefore improves the diagnosis and management of anemia; LHD% > 6.0% suggests iron deficiency [65, 66].

## 9. Summary

The official National Institutes of Health definition of a biomarker is “a characteristic that is objectively measured and evaluated as an indicator of normal biologic processes, pathogenic processes, or pharmacologic response to a therapeutic intervention” [67]. More generally, a biomarker is a laboratory measurement that can be used to measure the progress of disease or the effects of treatment, as the new RBC extended parameters can do and is proved in the published studies selected in the reference section. 

The percentage of hypochromic erythrocytes and the reticulocyte Hb content, reported by the modern analyzers, expand information at a cellular level:

(1) provide Hb content information on red cells;
(2) monitor changes in Hb incorporation into erythron;
(3) are more sensitive than indirect Biochemical measurements.
 Operational Efficiency:
  - rapid and automated;
  - rapid information to Clinicians of iron status;
  - faster response to changes from therapy;
  - financial justification.
 Aids clinicians in
  - assessing true status of iron;
  - detect functional iron deficiency, patients who can benefit from therapy;
  - differential diagnosis.

## 10. Conclusions

Several findings in the field of iron metabolism and erythropoiesis are modifying the traditional concepts on anemia. These findings point out the need for reliable diagnostic tests that are able to allow the better evaluation of the causes underlying apparently similar clinical conditions, implying a stronger collaboration between laboratory professionals and clinicians in order to optimize patient treatment.

Anemia is defined as a decrease concentration of Hb in the blood, cutoff depending on age and gender, but isolated Hb measurement has both low specificity and low sensitivity. The latter can be improved by including measures of iron-deficient erythropoiesis.

The biomarkers of hypochromia provide information about the iron supply and are reliable markers of iron restricted erythropoiesis in complex clinical situations.

An appropriate combination of laboratory tests gives evidence of iron depletion, reflects iron restricted red cells production, and so will help to establish a correct assessment of the iron status and thus the appropriate treatment.

## Figures and Tables

**Table 1:** The new biomarkers of hypochromia now reported by the analyzers of different manufacturers.

Parameter	Abbreviation (unit)	Company
Hypochromic RBC	Hypo (%)	Siemens
Reticulocyte Hb content	CHr (pg)	Siemens
Hypochromic RBC	%HPO (%)	Abbott
Reticulocyte Hb content	MCHr (pg)	Abbott
Hypochromic RBC	Hypo He (%)	Sysmex
Reticulocyte Hb equivalent	Ret He (pg)	Sysmex
Low Hb density	LHD (%)	Beckman Coulter

RBC: red blood cells; Hb: hemoglobin.

## References

[1] Swinkels DW, Janssen MCH, Bergmans J, Marx JJM (2006). Hereditary hemochromatosis: genetic complexity and new diagnostic approaches. *Clinical Chemistry*.

[2] Ganz T, Nemeth E (2009). Iron sequestration and anemia of inflammation. *Seminars in Hematology*.

[3] Fleming RE, Bacon BR (2005). Orchestration of iron homeostasis. *The New England Journal of Medicine*.

[4] Weiss G, Goodnough LT (2005). Anemia of chronic disease. *The New England Journal of Medicine*.

[5] Goodnough LT, Skikne B, Brugnara C (2000). Erythropoietin, iron, and erythropoiesis. *Blood*.

[6] Mast A (2001). The clinical utility of peripheral blood tests in the diagnosis of iron deficiency anemia. *Bloodline*.

[7] Coyne D (2006). Iron indices: what do they really mean?. *Kidney International Supplements*.

[8] Kalantar-Zadeh K, Rodriguez RA, Humphreys MH (2004). Association between serum ferritin and measures of inflammation, nutrition and iron in haemodialysis patients. *Nephrology Dialysis Transplantation*.

[9] Beerenhout C, Bekers O, Kooman JP, van der Sande FM, Leunissen KML (2002). A comparison between the soluble transferrin receptor, transferrin saturation and serum ferritin as markers of iron state in hemodialysis patients. *Nephron*.

[10] Skikne BS (2008). Serum transferrin receptor. *The American Journal of Hematology*.

[11] Weiss G (2009). Iron metabolism in the anemia of chronic disease. *Biochimica et Biophysica Acta*.

[12] Lankhorst CE, Wish JB (2010). Anemia in renal disease: diagnosis and management. *Blood Reviews*.

[13] Cavill I, Macdougall IC (1993). Functional iron deficiency. *Blood*.

[14] Macdougall IC (1995). Poor response to erythropoietin: practical guidelines on investigation and management. *Nephrology Dialysis Transplantation*.

[15] Zager RA, Johnson ACM, Hanson SY, Wasse H (2002). Parenteral iron formulations: a comparative toxicologic analysis and mechanisms of cell injury. *The American Journal of Kidney Diseases*.

[16] Wish JB (2006). Assessing iron status: beyond serum ferritin and transferrin saturation. *Clinical Journal of the American Society of Nephrology*.

[17] Katodritou E, Zervas K, Terpos E, Brugnara C (2008). Use of erythropoiesis stimulating agents and intravenous iron for cancer and treatment-related anaemia: the need for predictors and indicators of effectiveness has not abated. *British Journal of Haematology*.

[18] Locatelli F, Aljama P, Bárány P (2004). Revised European best practice guidelines for the management of anaemia in patients with chronic renal failure. *Nephrology Dialysis Transplantation*.

[19] Buttarello M, Plebani M (2008). Automated blood cell counts: state of the art. *The American Journal of Clinical Pathology*.

[20] Higgins JM, Mahadevan L (2010). Physiological and pathological population dynamics of circulating human red blood cells. *Proceedings of the National Academy of Sciences of the United States of America*.

[21] Cullen P, Söffker J, Höpfl M (1999). Hypochromic red cells and reticulocyte haemglobin content as markers of iron-deficient erythropoiesis in patients undergoing chronic haemodialysis. *Nephrology Dialysis Transplantation*.

[22] Thomas C, Thomas L (2002). Biochemical markers and hematologic indices in the diagnosis of functional iron deficiency. *Clinical Chemistry*.

[23] Mast AE, Blinder MA, Lu Q, Flax S, Dietzen DJ (2002). Clinical utility of the reticulocyte hemoglobin content in the diagnosis of iron deficiency. *Blood*.

[24] Brugnara C (2003). Iron deficiency and erythropoiesis: new diagnostic approaches. *Clinical Chemistry*.

[25] Macdougall IC (1998). Merits of percentage hypochromic red cells as a marker of functional iron deficiency. *Nephrology Dialysis Transplantation*.

[26] Bovy C, Gothot A, Krzesinski JM, Beguin Y (2005). Mature erythrocyte indices: new markers of iron availability. *Haematologica*.

[27] Bovy C, Gothot A, Delanaye P, Warling X, Krzesinski JM, Beguin Y (2007). Mature erythrocyte parameters as new markers of functional iron deficiency in haemodialysis: sensitivity and specificity. *Nephrology Dialysis Transplantation*.

[28] Kotisaari S, Romppanen J, Penttilä I, Punnonen K (2002). The Advia 120 red blood cell and reticulocyte indices are useful in diagnosis of iron-deficiency anemia. *European Journal of Haematology*.

[29] (2006). National kidney foundation, kidney disease outcomes quality initiative NKF-K/DOQI clinical practice guideline and clinical practice recommendations for anemia in chronic kidney disease. *The American Journal of Kidney Disease*.

[30] Locatelli F, Covic A, Eckardt KU, Wiecek A, Vanholder R (2009). Anaemia management in patients with chronic kidney disease: a position statement by the anaemia working group of European renal best practice (ERBP). *Nephrology Dialysis Transplantation*.

[31] Goodnough LT, Nemeth E, Ganz T (2010). Detection, evaluation, and management of iron-restricted erythropoiesis. *Blood*.

[32] Ervasti M, Kotisaari S, Heinonen S, Punnonen K (2007). Use of advanced red blood cell and reticulocyte indices improves the accuracy in diagnosing iron deficiency in pregnant women at term. *European Journal of Haematology*.

[33] Ervasti M, Kotisaari S, Sankilampi U, Heinonen S, Punnonen K (2007). The relationship between red blood cell and reticulocyte indices and serum markers of iron status in the cord blood of newborns. *Clinical Chemistry and Laboratory Medicine*.

[34] Suominen P, Punnonen K, Rajamäki A, Irjala K (1998). Serum transferrin receptor and transferrin receptor-ferritin index identify healthy subjects with subclinical iron deficits. *Blood*.

[35] Stoffman N, Brugnara C, Woods ER (2005). An algorithm using reticulocyte hemoglobin content (CHr) measurement in screening adolescents for iron deficiency. *Journal of Adolescent Health*.

[36] Ullrich C, Wu A, Armsby C (2005). Screening healthy infants for iron deficiency using reticulocyte hemoglobin content. *The Journal of the American Medical Association*.

[37] Shaker M, Jenkins P, Ullrich C, Brugnara C, Nghiem BT, Bernstein H (2009). An economic analysis of anemia prevention during infancy. *Journal of Pediatrics*.

[38] Ramakers C, van der Woude DAA, Verzijl JM, Pijnenborg JMA, van Wijk EM (2012). An added value for CHr and MCV in the diagnosis of iron deficency in postpartum anemic women. *International Journal of Laboratory Hematology*.

[39] Radtke H, Meyer T, Kalus U (2005). Rapid identification of iron deficiency in blood donors with red cell indexes provided by Advia 120. *Transfusion*.

[40] Nadarajan V, Sthaneshwar P, Eow GI (2008). Use of red blood cell indices for the identification of iron deficiency among blood donors. *Transfusion Medicine*.

[41] Semmelrock MJ, Raggam RB, Amrein K (2012). Reticulocyte hemoglobin content allows early and reliable detection of functional iron deficiency in blood donors. *Clinica Chimica Acta*.

[42] Kang SH, Kim HK, Ham CK, Lee DS, Cho HI (2008). Comparison of four hematology analyzers, CELL-DYN Sapphire, ADVIA 120, Coulter LH 750, and Sysmex XE-2100, in terms of clinical usefulness. *International Journal of Laboratory Hematology*.

[43] Ermens AAM, Hoffmann JJML, Krockenberger M, van Wijk EM (2012). New erythrocyte and reticulocyte parameters on CELL-DYN Sapphire; analytical and preanalytical aspects. *International Journal of Laboratory Hematology*.

[44] Hoffmann JJML, van den Broek NMA, Curvers J (2012). Reference intervals of extended erythrocyte and reticulocyte parameters. *Clinical Chemistry and Laboratory Medicine*.

[45] Costa O, van Moer G, Jochmans K, Jonckheer J, Damiaens S, de Waele M (2012). Reference values for new red blood cell and platelet parameters on the Abbott Diagnostics Cell-Dyn Sapphire. *Clinical Chemistry and Laboratory Medicine*.

[46] Buttarello M, Temporin V, Ceravolo R, Farina G, Bulian P (2004). The new reticulocyte parameter (RET-Y) of the Sysmex XE 2100: its use in the diagnosis and monitoring of posttreatment sideropenic anemia. *The American Journal of Clinical Pathology*.

[47] Canals C, Remacha AF, Sardá MP, Piazuelo JM, Royo MT, Angeles Romero M (2005). Clinical utility of the new Sysmex XE 2100 parameter—reticulocyte hemoglobin equivalent—in the diagnosis of anemia. *Haematologica*.

[48] Brugnara C, Schiller B, Moran J (2006). Reticulocyte hemoglobin equivalent (Ret He) and assessment of iron-deficient states. *Clinical and Laboratory Haematology*.

[49] Thomas C, Kirschbaum A, Boehm D, Thomas L (2006). The diagnostic plot: a concept for identifying different states of iron deficiency and monitoring the response to epoetin therapy. *Medical Oncology*.

[50] Thomas L, Franck S, Messinger M, Linssen J, Thomé M, Thomas C (2005). Reticulocyte hemoglobin measurement—comparison of two methods in the diagnosis of iron-restricted erythropoiesis. *Clinical Chemistry and Laboratory Medicine*.

[51] David O, Grillo A, Ceoloni B (2006). Analysis of red cell parameters on the Sysmex XE 2100 and ADVIA 120 in iron deficiency and in uraemic chronic disease. *Scandinavian Journal of Clinical and Laboratory Investigation*.

[52] Garzia M, Di Mario A, Ferraro E (2007). Reticulocyte hemoglobin equivalent: an indicator of reduced iron availability in chronic kidney diseases during erythropoietin therapy. *Laboratory Hematology*.

[53] Mast AE, Blinder MA, Dietzen DJ (2008). Reticulocyte hemoglobin content. *The American Journal of Hematology*.

[54] MacOni M, Cavalca L, Danise P, Cardarelli F, Brini M (2009). Erythrocyte and reticulocyte indices in iron deficiency in chronic kidney disease: comparison of two methods. *Scandinavian Journal of Clinical and Laboratory Investigation*.

[55] Miwa N, Akiba T, Kimata N (2010). Usefulness of measuring reticulocyte hemoglobin equivalent in the management of haemodialysis patients with iron deficiency. *International Journal of Laboratory Hematology*.

[56] Préfontaine G, Darveau M, Ahnadi C (2008). Reticulocyte hemoglobin content in 13 critically ill patients: a preliminary study. *Transfusion Alternatives in Transfusion Medicine*.

[57] Fernández R, Tubau I, Masip J, Muñoz L, Roig I, Artigas A (2010). Low reticulocyte hemoglobin content is associated with a higher blood transfusion rate in critically ill patients: a cohort study. *Anesthesiology*.

[58] Schoorl M, Snijders D, Schoorl M, Boersma WG, Bartels PCM (2012). Temporary impairment of reticulocyte hemoglobin content in subjects with community adquired pneumonia. *International Journal of Laboratory Hematology*.

[59] Urrechaga E, Borque L, Escanero JF (2009). Potential utility of the new sysmex XE 5000 red blood cell extended parameters in the study of disorders of iron metabolism. *Clinical Chemistry and Laboratory Medicine*.

[60] Urrechaga E, Borque L, Escanero JF (2012). Percentage of hypochromic erythrocytes as potential marker of iron availability. *Clinical Chemistry and Laboratory Medicine*.

[61] Urrechaga E, Borque L, Escanero JF Erythrocyte and reticulocyte indices in the assessment of erythropoiesis activity and iron availability. *International Journal of Laboratory Hematology*.

[62] Buttarello M, Pajola R, Novello E (2010). Diagnosis of iron deficiency in patients undergoing hemodialysis. *The American Journal of Clinical Pathology*.

[63] Urrechaga E (2010). The new mature red cell parameter, low haemoglobin density of the Beckman-Coulter LH750: clinical utility in the diagnosis of iron deficiency. *International Journal of Laboratory Hematology*.

[64] Urrechaga E, Borque L, Escanero JF (2010). Erythrocyte and reticulocyte indices on the LH 750 as potential markers of functional iron deficiency. *Anemia*.

[65] Urrechaga E, Borque L, Escanero JF (2012). Low Hemoglobin Density (LHD %) potential marker of iron availability. *International Journal of Laboratory Hematology*.

[66] Urrechaga E, Borque L, Escanero JF, Gööz M (2012). Assessing iron status in CKD patients: new Laboratory parameters. *Chronic Kidney Disease*.

[67] Ptolemy AS, Rifai N (2010). What is a biomarker? Research investments and lack of clinical integration necessitate a review of biomarker terminology and validation schema. *Scandinavian Journal of Clinical and Laboratory Investigation*.

